# Supplementary material for: Enhanced Catalytic Activity of Gold@Polydopamine Nanoreactors with Multi-compartment Structure Under NIR Irradiation
Source: Nanomicro Lett. 2019 Oct 10;11:83. doi: 10.1007/s40820-019-0314-9 (PMC7770829; doi:10.1007/s40820-019-0314-9)
Supplement: Supplementary file 1 — Supplementary material 1 (PDF 1029 kb) [file 40820_2019_314_MOESM1_ESM.pdf]

Supporting Information for

Enhanced Catalytic Activity of Gold@Polydopamine Nanoreactors with Multi-Compartment Structure under NIR Irradiation

Shilin Mei[1], Zdravko Kochovski[1], Rafael Roa[2], Sasa Gu[3], Xiaohui Xu[1], Hongtao Yu[1], Joachim Dzubiella[4, 5], Matthias Ballauff[1, 6], Yan Lu[1, 7, *]

[1]Soft Matter and Functional Materials, Helmholtz-Zentrum Berlin für Materialien und Energie, Hahn-Meitner Platz 1, 14109 Berlin, Germany

[2]Department of Applied Physics I, University of Málaga, 29071 Málaga, Spain

[3]College of Materials Science and Engineering, Nanjing Tech University, 210000 Nanjing, People's Republic of China

[4]Institute of Physics, University of Freiburg, 79104 Freiburg, Germany

[5]Simulation of Energy Materials, Helmholtz-Zentrum Berlin für Materialien und Energie, Hahn-Meitner Platz 1, 14109 Berlin, Germany

[6]Institut für Physik, Humboldt-Universität zu Berlin, Newtonstr. 15, 12489 Berlin, Germany

[7]Institute of Chemistry, University of Potsdam, 14476 Potsdam, Germany

*Corresponding author. E-mail: yan.lu@helmholtz-berlin.de (Yan Lu)

Supplementary Figures and Dissussion

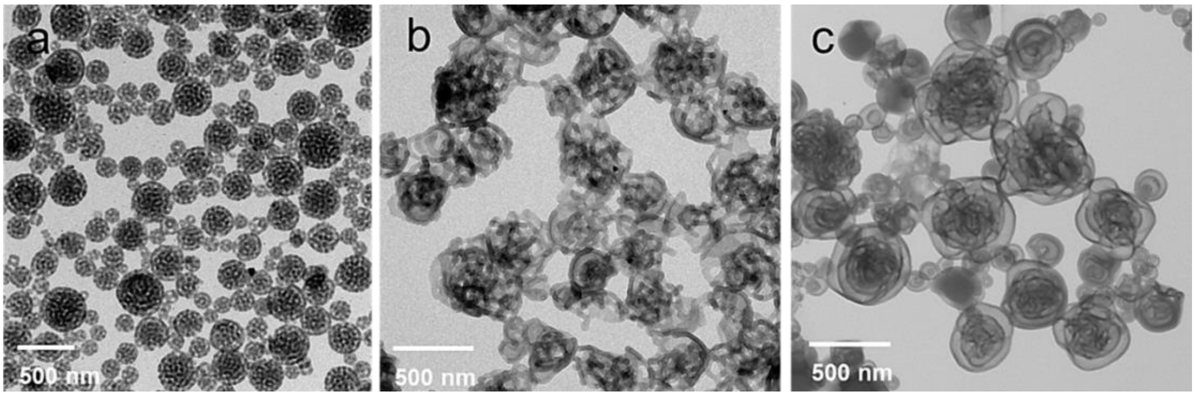

Fig. S1 Porous PS-P2VP particles with different swelling ratios. **a** M_n (P2VP) = 50000 g mol^{-1}, M_n (PS) = 16500 g mol^{-1}; **b** M_n (P2VP) = 10400 g mol^{-1}, M_n (PS) = 23600 g mol^{-1}; **c** M_n (P2VP) = 4800 g mol^{-1}, M_n (PS) = 26000 g mol^{-1}

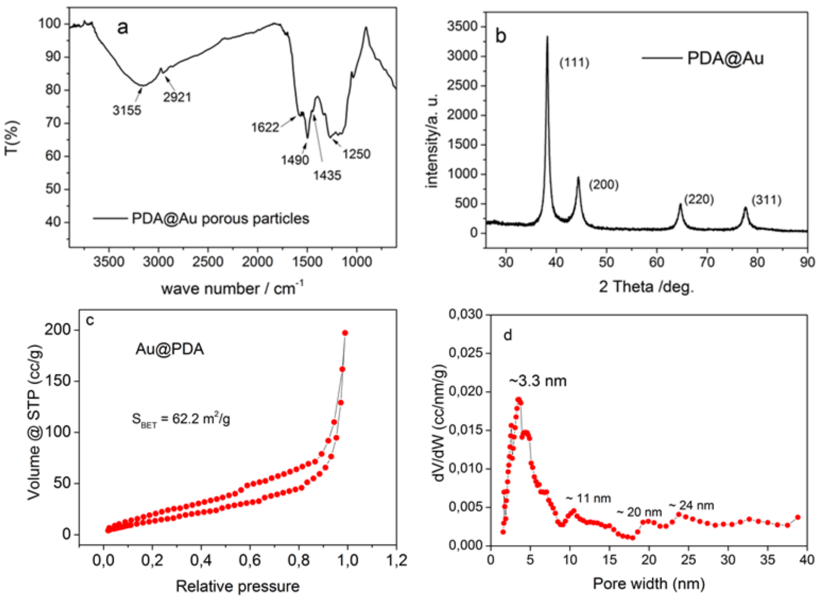

Fig. S2 a FTIR and **b** XRD spectra of the Au@PDA porous particles. **c** Nitrogen adsorption/desorption isotherms of the Au@PDA particles. **d** Pore size distribution curve

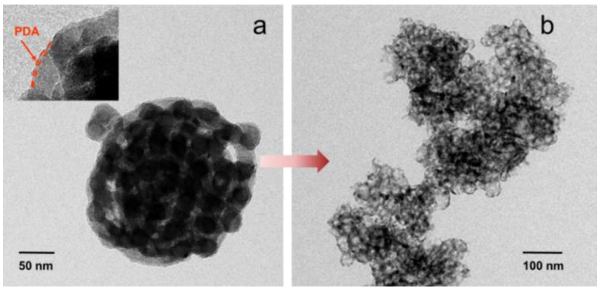

Fig. S3 a PDA@PS-P2VP porous particles produced from reaction time less than 4h: A very thin layer of PDA formed on the surface of the templates; **b** The distorted PDA particles after removal of the PS-P2VP template

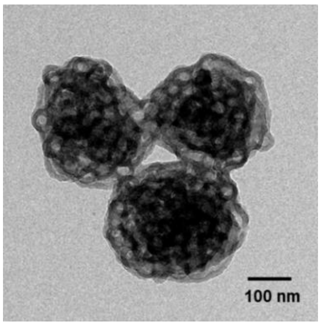

Fig. S4 PDA particles obtained from reaction for 11 h. The PDA thickness is ~14 nm

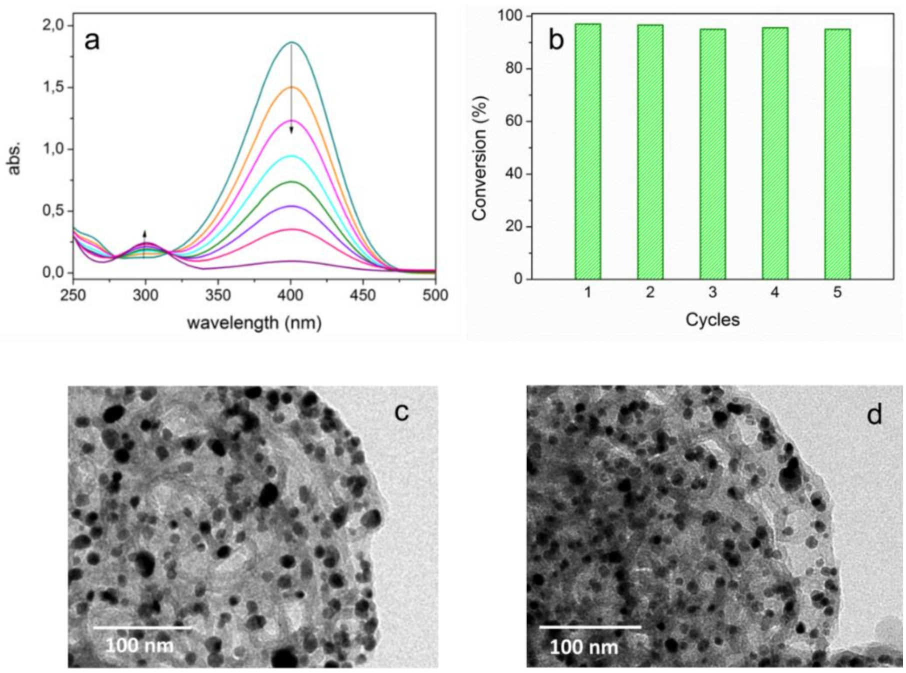

Fig. S5 **a** UV-vis spectra of the reduction of 4-nitrophenol by $NaBH_4$ using the Au@PDA particles as catalytic nanoreactors, **b** Conversion efficiency of the PDA@Au catalytic nanoreactors in 5 consecutive reaction cycles; TEM images of the PDA@Au particles before (c) and after (d) used in catalytic reduction reactions.

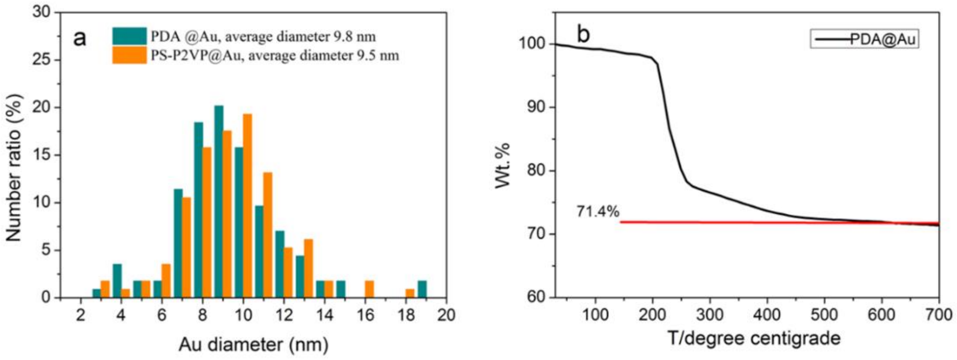

Fig. S6 Au NPs measured for the total surface area in the catalytic reactions.**a** Size distribution of the Au nanoparticles; **b** TGA measurement of the Au@PDA particles shows that the Au content is ~71.4%

Kinetic Analysis of the Catalytic Reduction of 4-Nitrophenol

The Langmuir-Hinshelwood kinetics [S1-S4] has been used for the mechanistic analysis of the catalytic activity. As shown in **Fig.** S7, 4-nitrophenol (Nip) is first reduced to 4-nitrosophenol and then to 4-hydroxylaminophenol (Hx). In the final step, Hx is reduced to 4-aminophenol (Amp) [S2]. The kinetic study follows a system of two coupled differential equations which describe the two steps of the reduction [S2-S4]:

$$-\frac{dc_{Nip}}{dt} = k_a S \frac{(K_{Nip})^n (c_{Nip})^n K_{BH4} c_{BH4}}{\left[1 + K_{Hx} c_{Hx} + (K_{Nip} c_{Nip})^n + K_{BH4} c_{BH4}\right]^2} = \left(\frac{dc_{Hx}}{dt}\right)_{source} \qquad \text{(S1)}$$

$$\frac{dc_{Hx}}{dt} = k_a S \frac{(K_{Nip})^n (c_{Nip})^n K_{BH4} c_{BH4}}{\left[1 + K_{Hx} c_{Hx} + (K_{Nip} c_{Nip})^n + K_{BH4} c_{BH4}\right]^2} - k_b S \frac{K_{Hx} c_{Hx} K_{BH4} c_{BH4}}{\left[1 + K_{Hx} c_{Hx} + (K_{Nip} c_{Nip})^n + K_{BH4} c_{BH4}\right]^2} \qquad \text{(S2)}$$

Here K_{Nip}, K_{Hx} and K_{BH4} are the Langmuir adsorption constants of the respective compounds, and k_a, k_b represent the reaction rate constants of step A and B, respectively. Equation (S1) describes the decay rate of 4-nitrophenol and the generation of Hx. Equation (S2) presents the generation and decay of the intermediate Hx. The concentration of 4-nitrophenol as the function of reaction time was then calculated by the numerical solution of Eqs. (S1) and (S2) as reported [S2-S4]

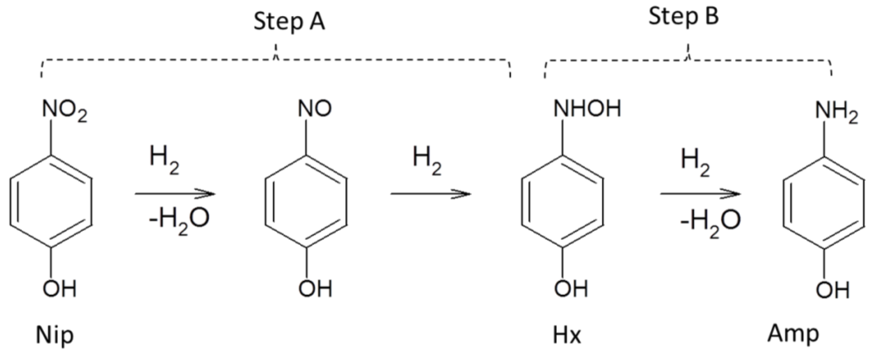

Fig. S7 Mechanism of the reduction of 4-Nip by metallic nanoparticles on the direct route

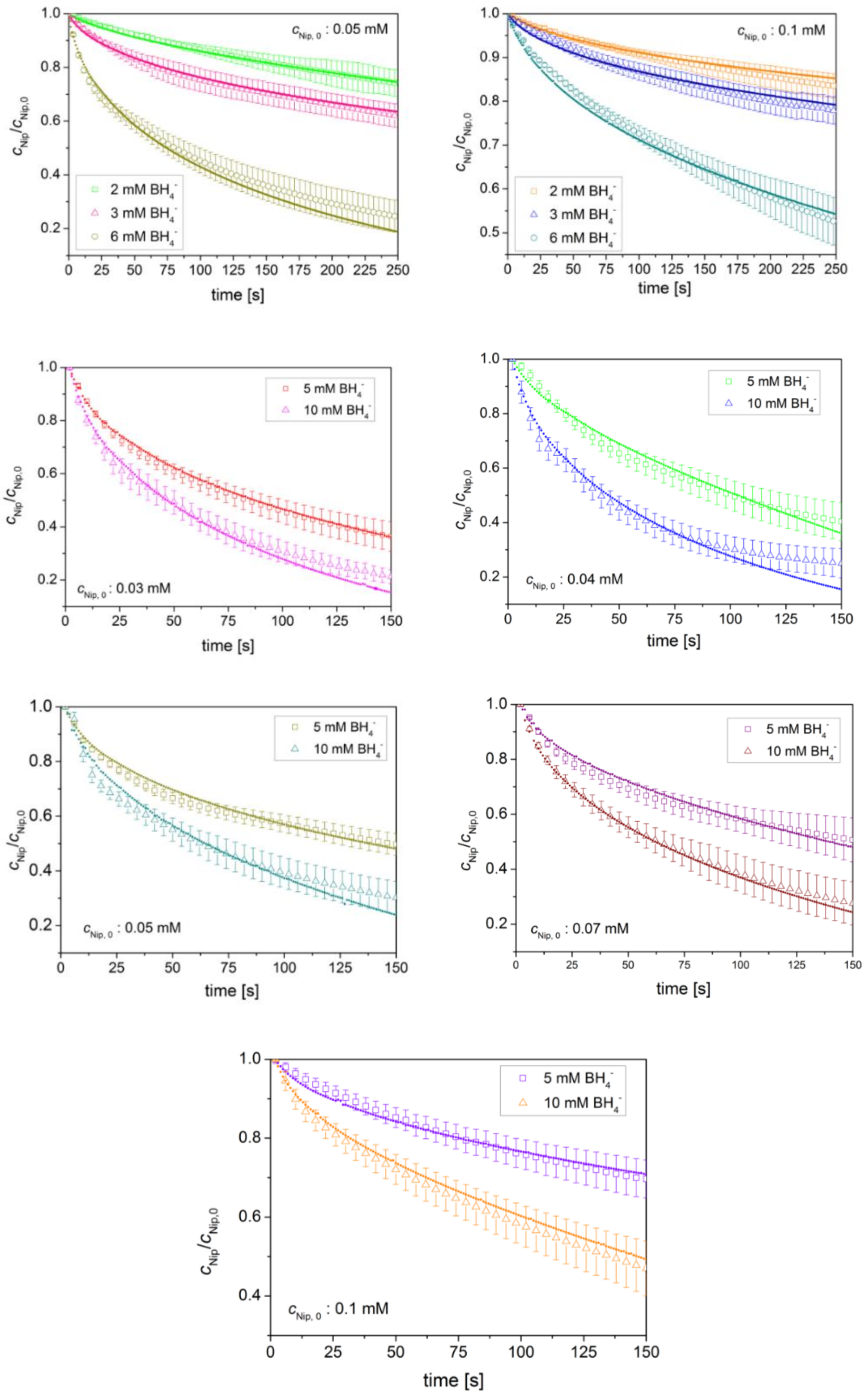

Fig. S8 Fit of the concentration of 4-nitrophenol as the function of time by the numerical solution of Eqs. (S1) and (S2). The solid lines refer to the fits by the kinetic model. The concentration of 4-nitrophenol was normalized to the respective starting concentration $c_{Nip,0}$. The experimental data have been taken from reactions at 20 °C (data points with error bars)

As shown in **Fig.** S8, the concentration of 4-nitrophenol normalized by respective initial concentration $c_{Nip,0}$ was plotted as the function of time with different initial

concentrations of 4-nitrophenol and BH_4^-. The solid lines are the fits by theory. From **Fig.** S8 it can be seen that the experimental data can be well fitted even when the conversion reaches 70%. This is the first time that full kinetic study has been approved to be applied for catalytic nanoreactor with complex nanostructure.

Theory for Surface Versus Diffusion Controlled Reactions

The total catalytic reaction time in a pseudo-unimolecular reaction, k_{app}^{-1}, is the sum of the time for the reactant 4-nitrophenol to diffuse to the gold nanoparticles in the Au@PDA nanoreactors, k_D^{-1}, and the time to get reduced by sodium borohydride $NaBH_4$ adjacent to a nanoparticle, k_S^{-1},

$$k_{app}^{-1} = k_D^{-1} + k_S^{-1} \quad \text{(S3)}$$

Likewise, the diffusion time has two contributions: the diffusion from the bulk to the Au@PDA nanoreactor, k_{D0}^{-1}, and the diffusion from the outer surface of the PDA shell to the surface of a gold nanoparticle, k_{Dg}^{-1}, i.e. $k_D^{-1} = k_{D0}^{-1} + k_{Dg}^{-1}$. Since the density of gold nanoparticles in the nanoreactor is very large (e.g., as shown in **Fig.** 3), according to theoretical study for nanoreactors [S5] the rate limiting step in the diffusion approach (i.e. the slowest time) is the mean time to reach the nanoreactor from the bulk. Thus, in the case of a fully diffusion-controlled reaction, the macroscopically observable rate is given by the Smoluchowski rate [S5]

$$k_D \approx 4\pi D_0 R_0 c_{NR} \quad \text{(S4)}$$

where $D_0 \sim 1$ nm^2 ns^{-1} is the diffusion coefficient of 4-nitrophenol in water, $R_0 \sim$ (100-200) nm is the outer radius of the nanoreactor, and $c_{NR} = 0.025$ mg mL^{-1} is the concentration of nanoreactors in solution. We estimate the molecular weight of our Au@PDA nanoreactors as:

$$MW_{NR} = V_{NR}\phi_{NR}\rho_{NR} \quad \text{(S5)}$$

being $V_{NR} = (4\pi/3)R_0^3$ the nanoreactor volume, ϕ_{NR} the fraction of empty space in the porous hybrid nanoreactors, which we estimate to be 0.5 (based on analysis of tomography, **Fig.** 5 in the main manuscript), and ρ_{NR} =1.5 g cm^{-3} the mass density of the nanoreactor which we assume to be equal to the mass density of condensed PDA [S6]. Taking this into account, we find that the molar concentration of Au@PDA nanoreactors is $c_{NR} \sim 4 \cdot 10^{-12}$ M.

If we make an order of magnitude analysis, we conclude that $k_D^{-1} \sim 0.1 - 1$ s. From **Fig.** S9 and the other experimental data, we note that the apparent rate constant is, $k_{app} \sim 10^{-3} - 10^{-2}$ s^{-1}, which means that the total catalytic reaction time is

$k_{app}^{-1} \sim 100 - 1000\ s$ s. Since the diffusion time is approximately at least 2-3 orders of magnitude smaller than the total catalytic reaction time, using Eq. (S3) we clearly conclude that the reaction is surface-controlled.

We express the surface reaction rate, k_S, as

$$k_S = K_{vol} c_{Nip,g} \qquad \text{(S6)}$$

Where $c_{Nip,g}$ is the 4-nitrophenol concentration in the nanoreactor next to the gold nanoparticles, and $K_{vol} = k_{vol}\Delta V$, being k_{vol} the fraction per unit time of the 4-nitrophenol molecules that are reduced by $NaBH_4$, and ΔV the volume of the shell next to the gold nanoparticles where effectively the chemical reaction is happening. As a consequence, the surface reaction is directly proportional to the 4-nitrophenol and BH_4^- concentrations within the reactive volume. For large concentrations, a competition of both reactants for reactive sites on the metal surface would lead to a saturation and to a subsequent slowdown of the surface reaction rate.

We find such behavior in the total reaction rate, **Fig.** S9. The apparent rate, k_{app}, increases linearly with increasing BH_4^- concentration. The diminution of k_{app} with increasing 4-nitrophenol concentration is due to the nearly full coverage of the nanoparticles surface by 4-nitrophenol, which as well slows down the injection of electrons to the metal surface. If the reaction would be diffusion-controlled, a modification of the BH_4^- concentration would not affect the total rate (since the concentration of BH_4^- is in large excess and the bimolecular reaction can be effectively considered as pseudo-unimolecular). This fact supports our conclusion about the surface-controlled nature of the catalytic reaction.

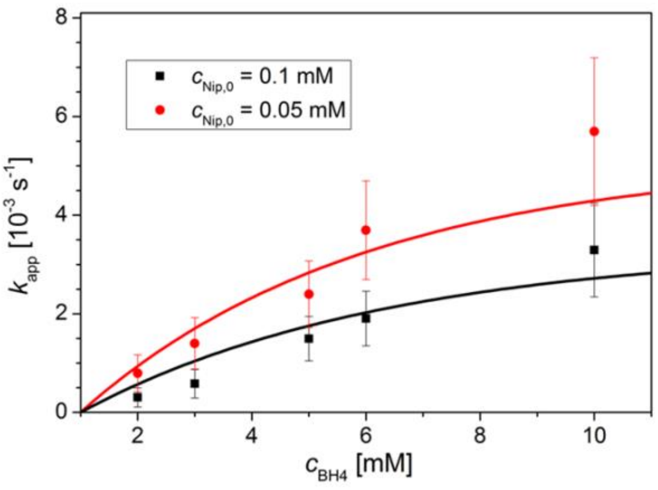

Fig. S9 Dependence of the apparent rate constant, k_{app}, with the borohydrate concentration for different initial 4-nitrophenol concentrations. The solid lines refer to the fit of the experimental data obtained using, e.g., Eq. (3a) in Ref. [S7]

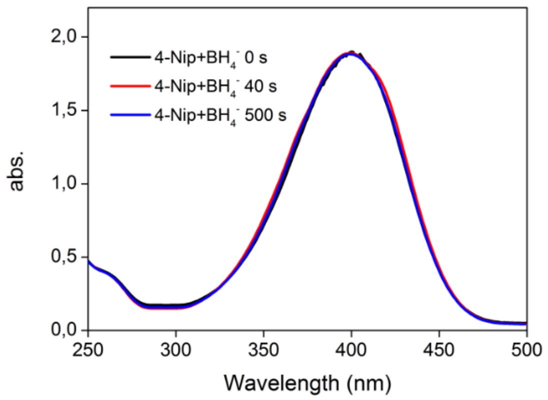

Fig. S10 Control experiment with only reactants of 4-nitrophenol and BH_4^- but without Au@PDA nanoreactors under NIR irradiation (808 nm, 3 W cm^{-2}, 500 S)

Supplementary References

[S1]S. Wunder, Y. Lu, M. Albrecht, M. Ballauff, Catalytic activity of faceted gold nanoparticles studied by a model reaction: evidence for substrate-induced surface restructuring. ACS Catal. **1**(8), 908-916 (2011). https://doi.org/10.1021/cs200208a

[S2]S. Gu, S. Wunder, Y. Lu, M. Ballauff, R. Fenger, K. Rademann, B. Jaquet, A. Zaccone, Kinetic analysis of the catalytic reduction of 4-nitrophenol by metallic nanoparticles. J. Phys. Chem. C **118**(32), 18618-18625 (2014). https://doi.org/10.1021/jp5060606.

[S3]S. Gu, Y. Lu, J. Kaiser, M. Albrecht, M. Balluaff, Kinetic analysis of the reduction of 4-nitrophenol catalyzed by Au/Pd nanoalloys immobilized in spherical polyelectrolyte brushes. Phys. Chem. Chem. Phys. **17**, 28137-28143 (2015). https://doi.org/10.1039/c5cp00519a

[S4]S. Gu, J. Kaiser, C. Marzum, A. Ott, Y. Lu, M. Balluaff, A. Zaccone, S. Barcikowski, P. Wagener, Ligand-free gold nanoparticles as a reference material for kinetic modelling of catalytic reduction of 4-nitrophenol. Catal. Lett. **145**(5), 1105-1112 (2015). https://doi.org/10.1007/s10562-015-1514-7

[S5]S. Angioletti-Uberti, Y. Lu, M. Ballauff, J. Dzubiella, Theory of solvation-controlled reactions in stimuli-responsive nanoreactors. J. Phys. Chem. C **119**(27), 15723-15730 (2015). https://doi.org/10.1021/acs.jpcc.5b03830

[S6]V. Ball, Determination of the extinction coefficient of “polydopamine” films obtained by using $NaIO_4$ as the oxidant. Mater. Chem. Phys. **186**(15), 546-551 (2017). https://doi.org/10.1016/j.matchemphys.2016.11.035

[S7]S. Wunder, F. Polzer, Y. Lu, Y. Mei, M. Ballauff, Kinetic analysis of catalytic reduction of 4-nitrophenol by metallic nanoparticles immobilized in spherical polyelectrolyte brushes. J. Phys. Chem. C **114**(19), 8814-8820 (2010). https://doi.org/10.1021/jp101125j
